# Pharmaco-utilisation and related costs of drugs used to treat schizophrenia and bipolar disorder in Italy: the IBIS study

**DOI:** 10.1186/s12888-014-0282-z

**Published:** 2014-10-14

**Authors:** Luca Degli Esposti, Diego Sangiorgi, Claudio Mencacci, Edoardo Spina, Carlotta Pasina, Marianna Alacqua, Flore la Tour

**Affiliations:** Health, Economics, and Outcomes Research, CliCon Srl, Via Salara 36, Ravenna, I-48121 Italy; Depression Unit, Neuroscience Department, Fatebenefratelli Hospital, Milan, Italy; Department of Clinical and Experimental Medicine, University of Messina, Messina, Italy; AstraZeneca Italy, Basiglio, Italy

**Keywords:** Antipsychotics, Real-world practice, Cost of illness, Pharmaco-utilisation, Schizophrenia, Bipolar disorder

## Abstract

**Background:**

Schizophrenia and bipolar disorder (BD) are psychiatric diseases that are commonly managed with antipsychotics. Treatment pathways are highly variable and no universal treatment guidelines are available. The primary objective of the *I*talian *B*urden of *I*llness in *S*chizophrenia and BD (IBIS) study was to describe pharmaco-utilisation of antipsychotic treatments and characteristics of patients affected by schizophrenia or BD. A secondary objective was to describe costs of illness for patients with schizophrenia or BD.

**Methods:**

IBIS was a multicentre, real-world, retrospective, observational cohort study based on data obtained from administrative databases of 16 Local Health Units in Italy (~7.5 million individuals). Patients with schizophrenia or BD ≥18 years of age treated with antipsychotics between 1 January 2008 and 31 December 2009 were included in the primary analysis. Pharmaco-utilisation data were gathered over a follow-up period of 12 months.

**Results:**

Patients with schizophrenia and BD received a wide variety of antipsychotic medications. The proportion of patients on antipsychotic monotherapy was 68% in patients with schizophrenia and 70% in patients with BD. In patients with schizophrenia, ~1/3 of patients receiving antipsychotic monotherapy also received mood stabilisers and/or antidepressants (34.7%) compared with over half of those on antipsychotic polytherapy (52.2%). In patients with BD, use of mood stabilisers and/or antidepressants was even higher; 76.9% of patients receiving antipsychotic monotherapy also received mood stabilisers and/or antidepressants compared with 85.5% of patients on antipsychotic polytherapy. Switch therapy was more frequent in patients with BD than in patients with schizophrenia, whereas add-on therapy was more frequent in patients with schizophrenia than in patients with BD. The mean total disease-related cost per patient per annum was higher in patients with schizophrenia (€4,157) than in patients with BD (€3,301). The number and cost of hospitalisations was higher in patients with BD, whereas the number and cost of nursing home stays was higher in patients with schizophrenia.

**Conclusion:**

Use of administrative databases has permitted retrieval of comprehensive information about therapeutic pathways, diagnostic history and costs in patients affected by schizophrenia or BD. A need for personalised treatment pathways has been described.

**Trial registration:**

clinicaltrials.gov: NCT01392482; first received June 29, 2011

## Background

Schizophrenia is a serious psychiatric disease with a high mortality risk compared with the general population (3.8-fold increase) [1] and is associated with greater comorbidity [2] and social exclusion [3]. With a prevalence of approximately four to seven per 1,000 persons [4], schizophrenia accounts for approximately 2.5% of total health care resources [5].

Bipolar disorder (BD) is the seventh most common global cause of disability for males and the eighth most common for females [6]. It affects approximately 1% of the world’s adult population and also has a high mortality rate [7]. Approximately 10–25% of patients suffering from BD attempt suicide, with a success rate of almost 0.4% per year [7,8].

Antipsychotic drugs are the mainstay of drug treatment of schizophrenia and BD, including first-generation agents such as haloperidol and second-generation agents such as olanzapine, risperidone and quetiapine, with many different drugs and regimens available globally [9–13]. An observational study in Italy, which examined patient characteristics and their pattern of care in 1,330 patients scheduled for discharge from acute psychiatric inpatient facilities, showed that the majority of patients, of which approximately half had schizophrenia or BD, were taking two or more drugs [14]. Treatment is tailored to an individual patient based on their clinical profile and comorbidities [15,16]; concomitant medication with anticonvulsants, mood stabilisers, anticholinergics, antidepressants and benzodiazepines is common [12,13]. Treatment pathways are highly variable and, as yet, no universal treatment guidelines are available [17–21]. Furthermore, application of the selected guidelines that do exist is highly dependent on the quality of the methodology used in their development, which is ultimately limited by financial resources [22]. The use of antipsychotic and antidepressant medication has increased in recent years [23] and this could be ascribed to a number of factors including the reduced stigma attached to mental health conditions, worsening economic conditions and the occurrence of natural disasters [23,24].

The *I*talian *B*urden of *I*llness in *S*chizophrenia and BD (IBIS) study was conducted to describe the pharmaco-utilisation of antipsychotic treatments in clinical practice, the characteristics of patients using these drugs and the costs associated with schizophrenia and BD to the Italian health service. At present, there are no studies in Italy showing the economic impact of these conditions.

The primary objective of the IBIS study was to describe the pharmaco-utilisation of antipsychotics (including typical and atypical) and the characteristics of patients affected by schizophrenia or BD in Italy. A secondary objective was to describe the costs of illness for patients with schizophrenia or BD. A further secondary objective, to describe treatment adherence and costs in patients who switched from quetiapine immediate release to quetiapine extended release, will be reported separately.

## Methods

### Study design and data sources

IBIS (ClinicalTrials.gov Identifier: NCT01392482) was a multicentre, real-world, retrospective, observational, cohort study of pharmaco-utilisation data obtained from the administrative databases of Local Health Units (LHUs) in Italy for patients with schizophrenia or BD.

Patients were enrolled from the administrative databases of 16 LHUs (approximately 7.5 million health-assisted individuals). As a point of delivery for the Italian National Health System, each LHU has an information network that routinely measures expenditure related to the dispensing of medications to registered patients. Such databases are used conventionally to record the amounts that pharmacies are entitled to receive from the LHU in respect to medications reimbursable through public finances and dispensed free of charge. Prescriptions are attributed to each individual patient via a personal health number.

The databases used were: (i) the *Health-assisted Subjects’ Database,* containing patients’ demographic data; (ii) the *Medications Prescription Database*, providing information for each prescription such as the prescribing physician’s number, the Anatomical-Therapeutic-Chemical (ATC) code of the drug purchased, the number of packs, the number of units per pack, the dosage, the unit cost per pack and the prescription date; (iii) the *Hospital Discharge Database*, which includes the date of the hospitalisation and discharge, admission and discharge wards, main and secondary discharge diagnosis, cost of the hospitalisation, based on Diagnosis Related Group (DRG) classification; (iv) the *Mental Health Information System*, which includes the diagnosis of the patient and nursing home accesses and psychiatric visits and related costs; (v) the *Specialist Outpatient Services Database* which includes information on outpatient services, dates and reimbursement costs, and (vi) the *No Charges Database* which includes information on free-of-charge services and dates.

The personal health number permitted electronic linking between all databases, allowing information on medication refills to be integrated with the patients’ date of birth, gender, any record of hospitalisation, diagnostic test and specialist visit. In order to guarantee patient privacy, each subject was assigned an anonymous, unambiguous numeric code. No identifiers related to patients were provided to the researchers. The study was approved by the local Ethics Committee in each participating LHU as follows: USL Arezzo, ASL Bergamo, ASL Bolzano, ASL Ferrara, ASL Firenze, USL Forlì, ASL Lecce, ASL Lecco, ASL Matera, ASL Monza e Brianza, ASL Piacenza, ASL Provincia di Milano 2, AUSL Ravenna, ASL Rimini, ASL Roma D, ASL Teramo, ASL RM/D, ASL Udine. The study was conducted in accordance with the Declaration of Helsinki and Good Publication Practice.

### Participants

Patients aged ≥18 years with a diagnosis of schizophrenia (International Classification of Disease [ICD]10 F20, ICD9 295.XX) or BD (ICD10 F30–F31, ICD9 296.XX excluding 296.2 and 296.3, depression) were enrolled. The phase of BD was established using the ICD code. The diagnoses were identified from two separate databases: the *Mental Health Information System* and the *Hospital Discharge Database* (primary or secondary diagnoses) during the period preceding the enrolment date. The use of hospital discharge records allowed inclusion of patients who did not access the Mental Health Department, but who developed a recurrence and were therefore hospitalised. Patients must have been registered with the LHU for the year before enrolment until 31 December 2010 or until they died, if earlier. Patients were enrolled regardless of how long they had received treatment. Patients who were transferred to another LHU during the period of observation were excluded, as were patients affected by schizo-affective disorders.

Patients were enrolled if they had filled at least one prescription for antipsychotics (ATC code N05A) between 1 January 2008 and 31 December 2009 (enrolment period). The enrolment date was defined as the first date on which a patient filled a prescription for an antipsychotic (the index date). Patients were characterised with regard to age, sex, diagnosis, treatments received and comorbidities during the year prior to enrolment.

### Pharmaco-utilisation

Starting from the index date, patients were followed for 1 year and pharmaco-utilisation data collected from the *Medications Prescription Database*, including the proportion of patients receiving an antipsychotic (ATC code: N05A); the proportion receiving concomitant central nervous system drugs (ATC codes: other N) (special attention was given to mood stabilisers [ATC code N03A] and antidepressants [ATC code: N06A]; lithium [ATC code N05AN] was considered as a mood stabiliser); and the individual antipsychotics prescribed and the combinations used. Antipsychotic monotherapy was defined as a treatment with one antipsychotic medication during the 1-year follow-up period. Antipsychotic polytherapy was defined as treatment with more than one antipsychotic medication during the 1-year follow-up period including switch therapy, where the patient’s treatment was changed during the follow-up period, and add-on treatments where patients received two or more antipsychotics simultaneously.

### Cost of illness and resources consumption

The costs to the health system were retrieved from the *Mental Health Information System*, the *Medications Prescription Database*, the *Specialist Outpatient Services Database* and the *Hospital Discharge Database* and were classified as either disease-related or unrelated to schizophrenia and BD.

Disease-related costs considered all the services provided by Mental Health Departments, in particular residential access (nursing home, full day) and semi-residential access (nursing home, half day). Disease-related outpatient specialist services included functional assessment (global, segmental, monofunctional), graduated psycho-behavioural assessment, clinical psychological interviews, psychiatric evaluation, psychiatric evaluation for control, psychotherapy (individual, family, group) and psychodiagnostic examination. The approach proposed by Weiden *et al.* [25], which takes into account a wide range of psychiatric diagnosis codes, was used to ensure all related hospitalisations were identified. Costs for hospitalisations were derived from the DRG codes and for outpatient services, costs were retrieved from regional tariffs.

Other disease-related costs considered included antipsychotics and concomitant central nervous system drugs (again, special attention was given to mood stabilisers and antidepressants; the price at the time of purchase was used).

Unrelated costs included all direct healthcare costs not previously described (e.g. a cardiovascular hospitalisation, other drugs not belonging to the previously specified ATC N group).

## Results

### Participants

#### Schizophrenia

A total of 12,943 patients were included in the dataset for the primary analysis. The dataset included 7,457 patients with schizophrenia representing 58% of the study population. The mean age of patients with schizophrenia was 48 years (women 53 years; men 46 years) and 40% were female.

#### Bipolar disorder

The dataset included 5,486 patients with BD representing 42% of the study population. The mean age of patients with BD was 52 years (women 54 years; men 50 years) and 59% were female. In patients with BD, 44.3% of patients were in a mania phase, 23.5% in a mixed phase, 16.5% in a depressive phase and 15.8% were unspecified.

### Pharmaco-utilisation

**Figure 1:**
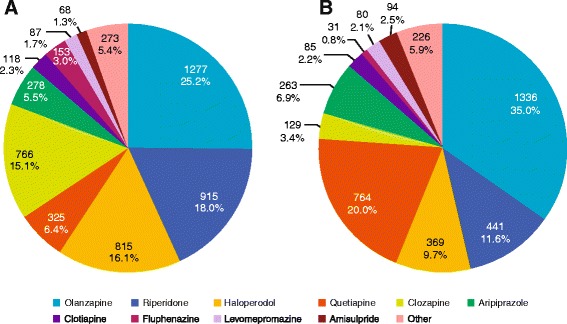
**Antipsychotic pharmaco-utilisation in patients treated with antipsychotic monotherapy for A) schizophrenia and B) bipolar disorder.**

**Figure 2:**
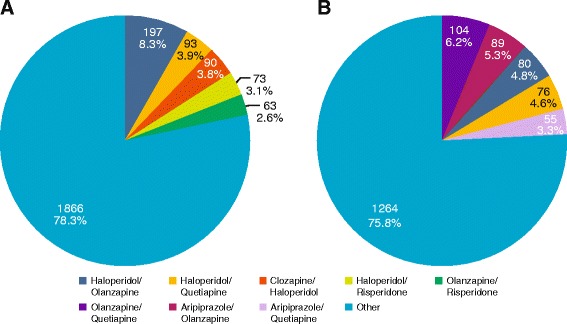
**Antipsychotic pharmaco-utilisation in patients treated with antipsychotic polytherapy for A) schizophrenia and B) bipolar disorder.**

**Figure 3:**
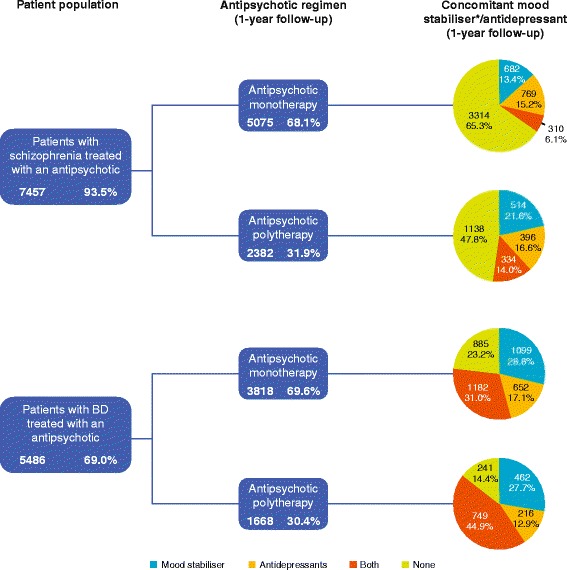
**Pharmaco-utilisation profile.** *Antiepileptic mood stabilisers + lithium.

A large number of prescription behaviours were observed (Figures 1, 2 and 3); across all patients with schizophrenia or BD, 24 different antipsychotics were used. In patients receiving antipsychotic monotherapy, more patients with BD received other drug classes, in addition to antipsychotics, than patients with schizophrenia (76.9% *versus* 34.7%) (Figure 3). For patients receiving polytherapy, 644 combinations were observed. The five most frequent combinations accounted for <25% of all combinations for both pathologies (Figure 2). In patients receiving antipsychotic polytherapy, more patients with BD received other drug classes, in addition to antipsychotics, than patients with schizophrenia (85.5% *versus* 52.2%). Switch therapy was more frequent in patients with BD (48%) than in patients with schizophrenia (31%), whereas add-on therapy was more frequent in patients with schizophrenia (69%) than in patients with BD (52%).

#### Schizophrenia

The proportion of patients with schizophrenia receiving treatment with a single antipsychotic (antipsychotic monotherapy) was 68% (Figure 3). The most commonly prescribed antipsychotic monotherapy for patients with schizophrenia was olanzapine (25%), followed by risperidone (18%) and haloperidol (16%) (Figure 1). Patients with schizophrenia receiving treatment with more than one antipsychotic (antipsychotic polytherapy) were more likely to receive other classes of pharmacological therapy such as mood stabilisers and/or antidepressants than those receiving antipsychotic monotherapy; in patients treated with antipsychotic polytherapy, 52.2% received mood stabilisers and/or antidepressants compared with only 34.7% treated with antipsychotic monotherapy (Figure 3).

#### Bipolar disorder

For patients with BD, the proportion on antipsychotic monotherapy was 70% (Figure 3). The most commonly prescribed antipsychotic monotherapy for patients with BD was olanzapine (35%), followed by quetiapine (20%) and risperidone (12%) (Figure 1). Patients with BD receiving antipsychotic polytherapy were also more likely to receive other therapies such as mood stabilisers and/or antidepressants than those receiving antipsychotic monotherapy; in patients treated with polytherapy, 85.5% received mood stabilisers and/or antidepressants compared with only 76.9% treated with antipsychotic monotherapy (Figure 3).

### Cost of illness and resources consumption

The mean total disease-related cost per patient per annum was higher in patients with schizophrenia (€4,157) than in patients with BD (€3,301) (Table 1). Treatment with antipsychotics accounted for approximately 25% of total disease-related costs for patients with schizophrenia, with nursing home costs and hospitalisations contributing 43% and 27% of total costs, respectively.

**Table 1:** **Cost of illness**

	**Schizophrenia**			**Bipolar disorder**		
	**€, mean**	***SD***	%*	**€, mean**	***SD***	%*
Antipsychotics (N05A)	1,018.81	*1,213.58*	24.5%	880.31	*1,000.24*	26.7%
Other drugs (CNS)	98.21	*228.52*	2.4%	264.87	*364.18*	8.0%
Ambulatory services	128.85	*372.64*	3.1%	114.08	*284.00*	3.5%
Hospitalisations	1,128.01	*3,779.11*	27.1%	1,542.01	*3,839.23*	46.7%
Nursing home	1,783.56	*6,173.32*	42.9%	499.76	*3,123.50*	15.1%
***TOTAL***	4,157.44			3,301.03		

*Percentage of total cost; SD, standard deviation; CNS, central nervous system.

Similarly, the cost of antipsychotics accounted for approximately 27% of total disease-related costs of BD; however, nearly half of the total cost was due to hospitalisations (46.7%) whereas nursing home costs accounted for only 15% (Table 1). These costs are reflected in the resource consumption for each disease: a higher proportion of patients with schizophrenia (18%) had a stay in a nursing home as compared with only just 8% of patients with BD. Furthermore, 26% of patients with BD were hospitalised whereas fewer patients with schizophrenia (18%) spent time in hospital (Table 2).

**Table 2:** **Resources consumption**

	**Schizophrenia (n = 7,457)**		**Bipolar disorder (n = 5,486)**	
Hospitalisations, pts (n,%, SD)	1,332	17.9 (0.4)	1,437	26.2 (0.6)
Hospitalisations, total	2,797		2,960	
Hospitalisations (mean ± SD)	0.38	0.01	0.54	0.01
Length of stay (days, total)	13,325		15,058	
Length of stay per hospital (days, mean ± SD)	18.2	34.1	18.9	29.9
Length of stay per pts (days, mean ± SD)	1.8	12.9	2.7	12.6
Nursing home, pts (n,%, SD)	1,368	18.3 (0.4)	441	8.0 (0.4)
Length of stay (days, mean ± SD)	15.5	51.9	4.2	25.2
Pts, ≥ 1 psychiatric visits, pts (n,%, SD)	4,266	57.2 (0.6)	3,207	58.5 (0.7)
Psychiatric visits (mean ± SD)	5.3	16.2	4.9	11.8

Pts, patients; SD, standard deviation.

## Discussion

Schizophrenia and BD are complex disorders requiring more targeted and personalised treatment approaches [15,16,18,26]. Treatment can be tailored to an individual patient according to their current symptoms, history of therapeutic and adverse effects, comorbidities and their expectations [15,16]. A number of clinical guidelines containing treatment algorithms are available to clinicians [17,27]. However, due to the complex nature of the management of these diseases and differences in the methodology used to create guidelines, recommendations are often conflicting [17–19]. Furthermore, our understanding of these disorders is constantly evolving and as a result guidelines can quickly become obsolete [18].

Real-world evidence is crucial for psychiatrists from a disease management perspective to aid the development of treatment guidelines, as large sample sizes representative of the general patient population can provide greater external validity than randomised controlled trials. The availability of population-wide databases is a preliminary and essential requirement for capturing real-world evidence and for the creation of an evidence-based research and management system [28,29]; although such data sources remain limited. To address this, epidemiological researchers have sought to utilise alternative data sources including medical and pharmacy claims databases similar to the administrative databases held by Italian LHUs [30–32].

Healthcare claims databases have many advantages for drug utilisation and economic research. These include a reliably representative picture of the clinical and treatment history of the monitored cohorts, the long duration of observation periods, the high generalisability of the results obtained and reasonable utilisation costs [33–35]. The large sample size and clinical setting may allow identification of previously undetected patterns of association that can be analysed in further studies.

The IBIS study showed that during the 1-year retrospective follow-up, patients with schizophrenia received a wide variety of antipsychotic medications, employed in many different combinations. This variety of combinations may be partially explained by a lack of Italian national guidelines and physicians tailoring therapy to fit the individual patient. A large number of the antipsychotic combinations used to treat patients with schizophrenia were inconsistent with those recommended in guidelines for the treatment of schizophrenia, such as those developed by the National Institute for Health and Care Excellence. For example, haloperidol is not recommended for use in combination with other antipsychotics due to an increased risk of QTc prolongation in the heart electrical cycle. In our study, as shown in Figure 2, haloperidol was used in some of the most common combinations. A substantial number of patients with schizophrenia received mood stabilisers and/or antidepressants in addition to their main antipsychotic therapy. Likewise, in patients with BD, a wide variety of antipsychotic medications were used, employed in various combinations and many also received mood stabilisers and/or antidepressants. Again, in a large number of cases, patients with BD were treated with antipsychotic combinations that are not recommended by current treatment guidelines for patients with BD. Based on our findings, we believe that there is a clear need for personalised treatment guidelines and recommendations, as although the current guidelines provide a valuable framework for pharmacological treatment, by their nature they are not appropriate for all patients. A more flexible approach would allow treatment to be tailored to patient characteristics and would more accurately reflect current clinical practice.

Differences in clinical management between the two diseases were evident. The cost of hospitalisations (e.g. emergency treatment) for patients with BD was considerably higher than in patients with schizophrenia, which may be indicative of a less stable patient population requiring acute/emergency care. This suggests there may be some scope to reduce costs by implementing changes to the clinical management of BD, possibly by early intervention to help prevent a bipolar episode escalating to a medical emergency. In another retrospective study, significant differences in mental health-related hospitalisations and costs were observed in patients with BD who received different formulations of the same drug, demonstrating how treatment choices can affect costs [36]. A recent systematic literature review of electronic databases, which analysed the cost of illness worldwide for BD, found that in-patient care or drugs costs were the main cost drivers in several studies [37]. Similarly, in a claims database analysis in a commercially insured population in the US, average claim costs per patient with schizophrenia were more than four times higher compared with a population without schizophrenia, and costs were mostly driven by in-patient charges in newly diagnosed patients [38]. Add-on therapy (simultaneous administration of more than one antipsychotic medication) was more frequent in the treatment of patients with schizophrenia than in patients with BD, which could be attributed to the availability of more well-defined diagnostic criteria for schizophrenia [13]. Indeed, in a retrospective study of US veteran databases, which described longitudinal antipsychotic prescribing patterns in schizophrenia, multiple prescribing pathways were observed and antipsychotic polypharmacy was commonly used as initial treatment [39]. The proportion of patients with BD in the manic phase of the disease was relatively high, possibly suggesting a selection bias through use of antipsychotics. The high ratio of patients with mania may affect the observed hospitalisation rate which may not be representative of the overall BD population. However, although antipsychotics are generally indicated for mania some can also be given as adjunctive treatment of bipolar depression or as part of maintenance therapy. Alternatively, as some of the databases used retrieved data from hospital records, patients in the mania phase are more likely to be included, as patients with mania are hospitalised more often than patients in the depressive phase [40–42]. However, the analysis also included data from non-hospitalised sources such as *Medications Prescription Database and the Specialist Outpatient Services Database.*

### Limitations

As this is a retrospective database study, associations between drug regimens and clinical outcomes can be assessed but causality cannot be inferred. Due to the collection methods used, the robustness of the data is dependent on the accuracy and extent of the information recorded in the database. In addition, the data are for the period 2009 to 2010 and so may not represent the most up to date practice. Administrative claims data in general are subject to potential coding errors and inconsistencies and may be affected by the limitations of clinical data. Moreover, in Italy diagnosis scales are rarely used and recorded. Other limitations of using administrative claims data are the exclusion of privately treated patients and ambiguity over the target indication of the therapy prescribed (some medications could have been taken for indications other than schizophrenia or BD, e.g. neuropathic pain, epilepsy, etc.).

## Conclusions

The use of administrative databases has permitted retrieval of comprehensive information about therapeutic pathways, diagnostic history and costs in patients affected by schizophrenia or BD providing psychiatrists and decision makers with information about pharmaco-utilisation of antipsychotic drugs. The wide variety of antipsychotic medications and combinations used to treat schizophrenia and BD observed in the IBIS study, reflects the evident need for personalised treatment. Current treatment guidelines are not suitable for all patients due to the complex nature of these diseases and their management. More targeted guidance that tailors treatment to patient characteristics would aid the management of these disorders.

## Abbreviations

ATC: Anatomical-Therapeutic-Chemical
BD: Bipolar disorder
DRG: Diagnosis Related Group
IBIS: *I*talian *B*urden of *I*llness in *S*chizophrenia and bipolar disorder
ICD: International Classification of Disease
LHU: Local Health Unit

## References

[1] Khan A, Faucett J, Morrison S, Brown WA (2013). Comparative mortality risk in adult patients with schizophrenia, depression, bipolar disorder, anxiety disorders, and attention-deficit/hyperactivity disorder participating in psychopharmacology clinical trials. JAMA Psychiatry.

[2] Laursen TM, Munk-Olsen T, Gasse C (2011). Chronic somatic comorbidity and excess mortality due to natural causes in persons with schizophrenia or bipolar affective disorder. PLoS ONE.

[3] Lavelle M, Healey PGT, McCabe R (2013). Is nonverbal communication disrupted in interactions involving patients with schizophrenia?. Schizophr Bull.

[4] Saha S, Chant D, Welham J, McGrath J (2005). A systematic review of the prevalence of schizophrenia. PLoS Med.

[5] Foster RH, Goa KL (1998). Risperidone. A pharmacoeconomic review of its use in schizophrenia. Pharmacoeconomics.

[6] World Health Organization: **The global burden of disease: 2004 update.** [http://www.who.int/healthinfo/global_burden_disease/2004_report_update/en/]

[7] Merikangas KR, Jin R, He JP, Kessler RC, Lee S, Sampson NA, Viana MC, Andrade LH, Hu C, Karam EG, Ladea M, Medina-Mora ME, Ono Y, Posada-Villa J, Sagar R, Wells JE, Zarkov Z (2011). Prevalence and correlates of bipolar spectrum disorder in the world mental health survey initiative. Arch Gen Psychiatry.

[8] Tondo L, Isacsson G, Baldessarini R (2003). Suicidal behaviour in bipolar disorder: risk and prevention. CNS Drugs.

[9] Verdoux H, Tournier M, Begaud B (2010). Antipsychotic prescribing trends: a review of pharmaco-epidemiological studies. Acta Psychiatr Scand.

[10] National Institute for Clinical Excellence: **Schizophrenia. Core interventions in the treatment and management of schizophrenia in adults in primary and secondary care (updated edition).** [http://guidance.nice.org.uk/CG82]

[11] National Institute of Clinical Excellence: **Bipolar disorder. The management of bipolar disorder in adults, children and adolescents, in primary and secondary care.** [http://guidance.nice.org.uk/CG38/Guidance/pdf/English]

[12] American Psychiatric Association: **Practice guideline for the treatment of patients with bipolar disorder.** [http://psychiatryonline.org/content.aspx?bookid=28&sectionid=1669577] 11958165

[13] American Psychiatric Association: **Practice guideline for the treatment of patients with schizophrenia second edition.** [http://psychiatryonline.org/content.aspx?bookid=28&sectionid=1665359]

[14] Preti A, Rucci P, Gigantesco A, Santone G, Picardi A, Miglio R, de Girolamo G (2009). Patterns of care in patients discharged from acute psychiatric inpatient facilities: a national survey in Italy. Soc Psychiatry Psychiatr Epidemiol.

[15] Kane JM, Correll CU (2010). Past and present progress in the pharmacologic treatment of schizophrenia. J Clin Psychiatry.

[16] Sobel S: **Effective personalized strategies for treating bipolar disorder.** [http://www.psychiatrictimes.com/bipolar-disorder/effective-personalized-strategies-treating-bipolar-disorder?pageNumber=2]

[17] Gaebel W, Riesbeck M, Wobrock T (2011). Schizophrenia guidelines across the world: a selective review and comparison. Int Rev Psychiatry.

[18] Nivoli AM, Colom F, Murru A, Pacchiarotti I, Castro-Loli P, Gonzalez-Pinto A, Fountoulakis KN, Vieta E (2011). New treatment guidelines for acute bipolar depression: a systematic review. J Affect Disord.

[19] Nivoli AM, Murru A, Goikolea JM, Crespo JM, Montes JM, Gonzalez-Pinto A, Garcia-Portilla P, Bobes J, Saiz-Ruiz J, Vieta E (2012). New treatment guidelines for acute bipolar mania: a critical review. J Affect Disorrd.

[20] Yatham LN, Kennedy SH, Parikh SV, Schaffer A, Beaulieu S, Alda M, O’Donovan C, Macqueen G, McIntyre RS, Sharma V, Ravindran A, Young LT, Milev R, Bond DJ, Frey BN, Goldstein BI, Lafer B, Birmaher B, Ha K, Nolen WA, Berk M (2013). Canadian Network for Mood and Anxiety Treatments (CANMAT) and International Society for Bipolar Disorders (ISBD) collaborative update of CANMAT guidelines for the management of patients with bipolar disorder: update 2013. Bipolar Disord.

[21] Goodwin GM (2009). Evidence-based guidelines for treating bipolar disorder: revised second edition–recommendations from the British Association for Psychopharmacology. J Psychopharmacol.

[22] Gaebel W, Weinmann S, Sartorius N, Rutz W, McIntyre JS (2005). Schizophrenia practice guidelines: international survey and comparison. Br J Psychiatry.

[23] Gualano MR, Bert F, Mannocci A, La Torre G, Zeppegno P, Siliquini R: **Consumption of antidepressants in Italy: recent trends and their significance for public health.** *Psychiatr Serv* 2014, [Epub ahead of print]. 10.1176/appi.ps.201300510 24981856

[24] Trifirò G, Italiano D, Alibrandi A, Sini G, Ferrajolo C, Capuano A, Spina E, Rossi A (2013). Effects of L’Aquila earthquake on the prescribing pattern of antidepressant and antipsychotic drugs. Int J Clin Pharm.

[25] Weiden PJ, Kozma C, Grogg A, Locklear J (2004). Partial compliance and risk of rehospitalization among California Medicaid patients with schizophrenia. Psychiatr Serv.

[26] Vieta E, Phillips ML (2007). Deconstructing bipolar disorder: a critical review of its diagnostic validity and a proposal for DSM-V and ICD-11. Schizophr Bull.

[27] Fountoulakis KN, Vieta E, Siamouli M, Valenti M, Magiria S, Oral T, Fresno D, Giannakopoulos P, Kaprinis GS (2007). Treatment of bipolar disorder: a complex treatment for a multi-faceted disorder. Ann Gen Psychiatry.

[28] Black N (1997). Developing high quality clinical databases. BMJ.

[29] Black N, Payne M (2002). Improving the use of clinical databases. BMJ.

[30] Hartzema AG, Racoosin JA, MaCurdy TE, Gibbs JM, Kelman JA (2011). Utilizing Medicare claims data for real-time drug safety evaluations:is it feasible?. Pharmacoepidemiol Drug Saf.

[31] Mitchell JB, Bubolz T, Paul JE, Pashos CL, Escarce JJ, Muhlbaier LH, Wiesman JM, Young WW, Epstein RS, Javitt JC (1994). Using Medicare claims for outcomes research. Med Care.

[32] Quam L, Ellis LB, Venus P, Clouse J, Taylor CG, Leatherman S (1993). Using claims data for epidemiologic research. The concordance of claims-based criteria with the medical record and patient survey for identifying a hypertensive population. Med Care.

[33] Riley GF (2009). Administrative and claims records as sources of health care cost data. Med Care.

[34] Birnbaum HG, Cremieux PY, Greenberg PE, LeLorier J, Ostrander JA, Venditti L (1999). Using healthcare claims data for outcomes research and pharmacoeconomic analyses. Pharmacoeconomics.

[35] Motheral BR, Fairman KA (1997). The use of claims databases for outcomes research: rationale, challenges, and strategies. Clin Ther.

[36] Locklear JC, Alemayehu B, Brody RS, Chavoshi S, Tunceli O, Kern D, Earley WR (2013). Treatment patterns, healthcare resource utilization and costs in patients with bipolar disorder, newly treated with extended release of immediate release quetiapine fumarate using US healthcare administrative claims data. Clin Ther.

[37] Kleine-Budde K, Touil E, Moock J, Bramesfield A, Kawohl W, Rössler W (2014). Cost of illness for bipolar disorder: a systematic review of the economic burden. Bipolar Disord.

[38] Fitch K, Iwasaki K, Villa KF (2014). Resource utilization and cost in a commercially insured population with schizophrenia. Am Health Drug Benefits.

[39] Goren JL, Meterko M, Williams S, Young GJ, Baker E, Chou C-H, Kilbourne AM, Bauer MS (2013). Antipsychotic prescribing pathways, polypharmacy, and clozapine use in treatment of schizophrenia. Psychiatr Serv.

[40] Al Jurdi RK, Schulberg HC, Greenberg RL, Kunik ME, Gildengers A, Sajatovic M, Mulsant BH, Young RC, GERI-BD Study Group (2012). Characteristics associated with inpatient versus outpatient status in older adults with bipolar disorder. J Geriatr Psychiatry Neurol.

[41] Fajutrao L, Locklear J, Priaulx J, Heyes A (2009). A systematic review of the evidence of the burden of bipolar disorder in Europe. Clin Pract Epidemiol Ment Health.

[42] Das Gupta R, Guest JF (2002). Annual cost of bipolar disorder to UK society. Br J Psychiatry.

